# TonEBP Promotes β-Cell Survival under ER Stress by Enhancing Autophagy

**DOI:** 10.3390/cells9091928

**Published:** 2020-08-20

**Authors:** Hyun Je Kang, Eun Jin Yoo, Hwan Hee Lee, Seung Min An, Hyun Park, Whaseon Lee-Kwon, Soo Youn Choi, Hyug Moo Kwon

**Affiliations:** School of Life Sciences, Ulsan National Institute of Science and Technology, Ulsan 44919, Korea; hjkang90@unist.ac.kr (H.J.K.); ejyoo89@unist.ac.kr (E.J.Y.); hheelee89@unist.ac.kr (H.H.L.); asm7615@unist.ac.kr (S.M.A.); skyline@unist.ac.kr (H.P.); wlee@unist.ac.kr (W.L.-K.)

**Keywords:** NFAT5, autophagy initiation, islet, FIP200, unfolded protein response, UPR

## Abstract

The endoplasmic reticulum (ER) stress response and autophagy are important cellular responses that determine cell fate and whose dysregulation is implicated in the perturbation of homeostasis and diseases. Tonicity-responsive enhancer-binding protein (TonEBP, also called NFAT5) is a pleiotropic stress protein that mediates both protective and pathological cellular responses. Here, we examined the role of TonEBP in β-cell survival under ER stress. We found that TonEBP increases β-cell survival under ER stress by enhancing autophagy. The level of TonEBP protein increased under ER stress due to a reduction in its degradation via the ubiquitin–proteasome pathway. In response to ER stress, TonEBP increased autophagosome formations and suppressed the accumulation of protein aggregates and β-cell death. The Rel-homology domain of TonEBP interacted with FIP200, which is essential for the initiation of autophagy, and was required for autophagy and cell survival upon exposure to ER stress. Mice in which *TonEBP* was specifically deleted in pancreatic endocrine progenitor cells exhibited defective glucose homeostasis and a loss of islet mass. Taken together, these findings demonstrate that TonEBP protects against ER stress-induced β-cell death by enhancing autophagy.

## 1. Introduction

The endoplasmic reticulum (ER) is an important intracellular organelle for the synthesis, folding, and assembly of secreted and transmembrane proteins. ER function is disturbed in several physiological and pathological conditions, and this leads to ER stress, which is characterized by the accumulation and aggregation of unfolded and/or misfolded proteins in the ER [1,2]. Cells react to ER stress by initiating the unfolded protein response (UPR), which is an adaptive and cellular protective response that aims to reduce the accumulation of unfolded proteins and restore ER homeostasis. However, an insufficient UPR and/or persistent ER stress trigger cellular dysfunction and cell death, leading to human diseases [2,3,4]. Importantly, β-cells in pancreatic islets contain a highly developed ER to produce insulin and thus are vulnerable to ER stress [5]. ER stress and the UPR are being increasingly implicated in the dysfunction and loss of pancreatic β-cells associated with the development of type 1 and type 2 diabetes mellitus (DM) [6,7].

Autophagy is a conserved lysosomal degradation pathway that involves recognizing the material for autophagic degradation, isolating the material via autophagosome formation and fusing autophagosomes with lysosomes (autolysosome) to degrade the cargo, and is essential for cellular homeostasis and adaptations to stress [8,9]. Autophagy is reciprocally linked to ER stress in eukaryotic cells. In response to ER stress, autophagy is induced to remove misfolded/aggregated proteins and damaged organelles, thereby improving cellular function and cell survival [10]. Conversely, blockade of autophagy increases ER stress and cell death [10,11,12]. The interplay between ER stress and autophagy is implicated in the physiology of β-cells. Transgenic mice in which autophagy is perturbed in β-cells exhibit increased β-cell death, decreased β-cell proliferation, and thus a reduced β-cell mass [13]. Autophagy-deficient β-cells display a distention of the ER, accumulation of polyubiquitinated proteins, and increased formation of large intracellular aggregates, which are related to the susceptibility of β-cells to ER stress [1], indicating that autophagy is essential for the survival and function of β-cells. However, the cellular mechanism underlying the interplay between autophagy and ER stress remains to be fully elucidated.

Tonicity-responsive enhancer-binding protein (TonEBP), which is also known as nuclear factor of activated T-cells 5 (NFAT5), was initially identified as a transcriptional regulator of the cellular response to hypertonic stress in the renal medulla [14,15,16]. Numerous studies have revealed that TonEBP is a pleiotropic stress protein that is involved in the response not only to hypertonicity but also to various types of stress, and leads to physiological or pathological consequences depending on the context [17]. The induction and activation of TonEBP in response to autoimmune and metabolic stresses are implicated in immunometabolic diseases such as rheumatoid arthritis [18,19], atherosclerosis [20], hepatocellular carcinoma [21], obesity [22], and DM [22,23]. By contrast, TonEBP-mediated responses to hypertonicity [16,24,25,26,27], bacterial infection [28,29,30], and genotoxic stress [31] have protective or homeostatic functions. Although the function of TonEBP in the responses to a range of cellular stresses is well-established, its role in the determination of cell fate under ER stress remains to be elucidated. Here, we explored the potential role of TonEBP in β-cell survival under ER stress. We found that TonEBP enhances ER stress-induced autophagy in β-cells and thereby increases β-cell survival. These findings suggest that TonEBP protects against ER stress-induced β-cell death.

## 2. Materials and Methods

### 2.1. Cells and Reagents

The MIN6-M9 mouse pancreatic β-cell line was provided by Prof. Seino (Kobe University, Kobe, Japan). MIN6-M9 and human embryonic kidney 293 (HEK293; ATCC CRL-1573) cells were cultured in Dulbecco’s Modified Eagle’s medium supplemented with 10% fetal bovine serum (Thermo Fisher Scientific, Waltham, MA, USA) and penicillin/streptomycin (100 U/mL and 100 μg/mL, respectively; GE Healthcare Life Sciences, Boston, UT, USA). Cells were maintained at 37 °C in an incubator containing 5% CO_2_. The antibodies used for immunoblotting or immunoprecipitation were obtained from various companies. Antibodies against ubiquitin (SantaCruz Biotechnology, Santa Cruz, CA, USA), BiP (Abcam, Cambridge, UK), LC3 (Cell Signaling Technology, Beverly, MA, USA), Hsc70 (Rockland, Gilbertsville, PA, USA), FIP200 (Abcam), Flag (Sigma-Aldrich, Saint Louis, MO, USA), Myc (Cell Signaling Technology), and HA (Sigma-Aldrich) were used. The primary antibodies were detected with horseradish peroxidase-conjugated mouse, rabbit, or goat secondary antibodies (Thermo Fisher Scientific) and Alexa Fluor 488-, 568-, or 633-conjugated secondary antibodies (Invitrogen, Carlsbad, CA, USA).

### 2.2. Transfection

Cells were transfected with the same concentrations of scrambled or gene-targeted siRNAs for 24 h using Lipofectamine RNAimax (Invitrogen) according to the manufacturer’s instructions. All siRNA duplexes were purchased from Integrated DNA Technologies (Coralville, IA, USA). All plasmids were purified using an endotoxin-free purification system (Qiagen, Valencia, CA, USA) and transfected into cells using Lipofectamine 2000 (Invitrogen) for 24 h. Transfected cells were then cultured in fresh complete medium and were analyzed as indicated in the figure legends.

### 2.3. Cell Viability Assay

MIN6-M9 cells were plated in triplicate in 96-well plates, and treated with brefeldin A (20 μM; Sigma-Aldrich), or tunicamycin (1 μg/mL; Sigma-Aldrich) for 24 h. Lactate dehydrogenase (LDH) release (Clontech, Mountain View, CA, USA) and 3-(4,5-dimethylthiazol-2-yl)-2,5-diphenyltetrazolium bromide (MTT) reduction (Sigma-Aldrich) were used to measure cell viability according to the manufacturer’s protocol. The LDH release was calculated as a percentage using the following formula: percentage = (sample–spontaneous release/maximum release−spontaneous release) × 100. MTT reduction was calculated as a percentage of the respective controls.

### 2.4. Immunofluorescence Staining

Cells were plated on LabTek chamber slides (Thermo Fisher Scientific), incubated for 24 h, and treated with brefeldin A (20 μM) or tunicamycin (1 μg/mL) with or without a pretreatment of LY294002 (10 μM; Sigma-Aldrich) or chloroquine (10 μM; Sigma-Aldrich) for 1 h. Cells were treated for 4 h or 6 h to detect LC3 puncta and 24 h to detect ubiquitination and BiP, and fixed with 100% methanol at 20 °C for 30 min. Fixed cells were stained with anti-ubiquitin, anti-BiP, and anti-LC3 primary antibodies overnight at 4 °C, washed with 0.05% Triton X-100, and then stained with Alexa Fluor-conjugated secondary antibodies for 1 h. The stained cells were mounted. Pancreatic tissues were fixed overnight in 4% paraformaldehyde and embedded in paraffin. The paraffin sections were deparaffinized and dehydrated. Immunohistochemistry was performed using anti-BiP antibodies under optimized conditions. Images were acquired using an Olympus FV1000 confocal fluorescence microscope.

### 2.5. Immunoblotting

Cells were treated with brefeldin A (20 μM) or tunicamycin (1 μg/mL) for 1 h to detect ubiquitination and 6 h to detect the levels of proteins. Cells were washed twice with cold phosphate-buffered saline (PBS) and lysed in RIPA buffer (0.01 M Tris, pH 7.4, 0.15 M NaCl, 0.001 M EDTA, 0.001 M EGTA, and 1% Triton X-100; all from Sigma-Aldrich) containing 0.002 M phenylmethylsulfonyl fluoride (PMSF; Sigma-Aldrich) and protease inhibitors (Roche, Rotkreuz, Switzerland). After the centrifugation of the lysate, the supernatant was used for an immunoblot analysis. The protein concentration was measured with the BCA Protein Assay System (Pierce Biotechnology, Rockford, IL, USA). Proteins were denatured in Laemmli buffer. Equal amounts of each sample were separated on SDS-polyacrylamide gels and then transferred to polyvinylidene difluoride (PVDF) membranes. The membranes were blocked, incubated with primary antibodies, and washed using PBS supplemented with 0.05% (*v*/*v*) Tween-20 and 5% (*w*/*v*) nonfat dry milk. Anti-TonEBP [14], anti-LC3, anti-FIP200, anti-Flag, anti-Myc, anti-HA, and anti-BiP primary antibodies were used for immunoblotting. An anti-Hsc70 primary antibody was used as a loading control. Horseradish peroxidase-conjugated secondary antibodies were used for detection. Reactive bands were detected by chemiluminescence using the ImageQuant LAS 4000 imaging system (GE Healthcare Life Sciences).

### 2.6. Immunoprecipitation

Cells were washed three times with ice-cold PBS and then incubated with RIPA buffer in a tube on ice to prepare the total cell lysates. The lysates were incubated with anti-TonEBP, anti-Myc, anti-Flag, and anti-HA antibodies overnight at 4 °C under rotary agitation and then with Protein A/G Sepharose beads (GE Healthcare Sciences) for 2 h at 4 °C under rotary agitation. Bead–antibody–antigen complexes were pelleted by centrifugation at 4 °C for 1 min and the supernatant was removed. Complexes were washed three times for 10 min with RIPA buffer at 4 °C, supplemented with a sample buffer, and boiled at 95 °C for 5 min. The samples were analyzed by immunoblotting.

### 2.7. Mice

All procedures involving live mice were carried out in accordance with the approved guidelines of the Institutional Animal Care and Use Committee of the Ulsan National Institute of Science and Technology (UNISTACUC-16-08). All experiments used male C57BL/6 J mice. Mice carrying the loxP-targeted *TonEBP* gene (*TonEBP^fl/fl^*) have been described previously [32] and were provided by Dr. Neuhofer (Division of Nephrology and Rheumatology, Clinical Center Traunstein, D-83278 Traunstein, Germany). Neurog3-cre knock-in mice, known as Ngn3-cre mice, were obtained from Jackson Laboratories (Bar Harbor, ME, USA). The *TonEBP^fl/fl^* mice were crossed with Ngn3-cre mice to generate mice that lacked TonEBP in pancreatic endocrine progenitor cells. Age- and sex-matched littermates were used as controls in all experiments.

### 2.8. Statistical Analysis

Data are expressed as the mean + standard deviation or standard error of the mean. The statistical significance of the differences between the two conditions was estimated using an unpaired *t*-test. More than two conditions were compared using a one-way ANOVA and Tukey’s post-hoc test. A *p*-value < 0.05 was deemed significant. All statistical analyses were performed using GraphPad Prism 8.2 software (GraphPad, San Jose, CA, USA).

## 3. Results

### 3.1. TonEBP Suppresses the ER Stress-Induced Accumulation of Unfolded Proteins and β-Cell Death

**Figure 1:**
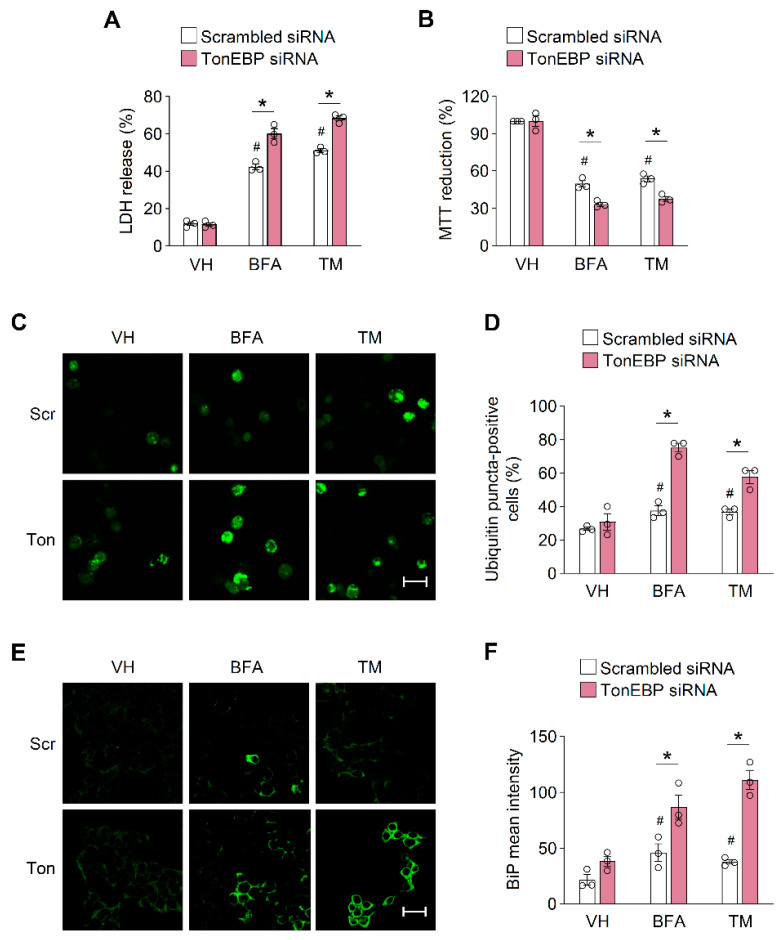
Tonicity-responsive enhancer-binding protein (TonEBP) prevents the accumulation of unfolded proteins. (**A**,**B**) MIN6-M9 cells were transfected with scrambled siRNA or TonEBP-targeted siRNA, and then treated with vehicle (VH), brefeldin A (BFA; 20 μM), or tunicamycin (TM; 1 μg/mL). Cell viability was assessed by the LDH release (**A**) and MTT reduction (**B**) after 24 h. (**C**) Cells were transfected with scrambled siRNA (Scr) or TonEBP-targeting siRNA (Ton) and then treated as above. Ubiquitin was visualized with an anti-ubiquitin antibody by immunostaining. (**D**) Percent of ubiquitin puncta positive cells were counted from 100 cells in each group. (**E**) Cells were transfected and treated as in (**C**). BiP was detected with an anti-BiP antibody by immunostaining. (**F**) Percent of BiP positive cells were counted from 100 cells in each group. VH, vehicle. Data (mean + SD) were from three independent experiments (*n* = 3) each with more than three replicates. # *p* < 0.05 vs. scrambled siRNA-VH. * *p* < 0.05 ((**A**,**B**); unpaired *t*-test, (**D**,**F**); one-way ANOVA). Scale bars, 50 μm (**C**,**E**).

To investigate the role of TonEBP in β-cell survival under ER stress, we first examined whether the siRNA-mediated depletion of TonEBP affects cell death triggered by agents that induce ER stress, namely, brefeldin A (BFA) and tunicamycin (TM). MIN6-M9 mouse β-cells transfected with scrambled siRNA or TonEBP-targeted siRNA were incubated with 20 μM BFA or 1 μg/mL TM for 24 h followed by an LDH or MTT assay. Treatment with these agents reduced the viability of β-cells, and the TonEBP depletion dramatically increased the cell death induced by ER stress inducers, BFA and TM (Figure 1A,B). Conversely, TonEBP overexpression attenuated cell death induced by BFA, but not by TM (Figure S1A). These results suggest that TonEBP increases β-cell survival under ER stress.

We next investigated the mechanism by which TonEBP determines β-cell fate under ER stress. Ubiquitinated and unfolded proteins commonly accumulate in response to ER stress [33,34] and this triggers the activation of the UPR, which leads to the removal of these proteins [2]. However, a prolonged activation of the UPR leads to cell death under persistent ER stress [35]. We first examined whether TonEBP modulates the accumulation of ubiquitinated proteins by performing an immunofluorescence analysis of ubiquitin. The formation of ubiquitin foci markedly increased under ER stress, and TonEBP depletion enhanced the formation of ubiquitin foci in response to ER stress inducers (Figure 1C,D). We next examined the induction of BiP (also called GRP78), which is a molecular indicator of ER stress and UPR activation [36]. TonEBP depletion increased the number of BiP-positive cells observed after 24 h treatment with ER stressors (Figure 1E,F). Additionally, TonEBP depletion did not affect the protein and mRNA expression of BiP (Figure S1B,C), and the mRNA expression of ER stress-related genes *dit3*, *Atf4*, and *Ire1a* (Figure S1D–F) in a 4 h treatment with ER stressors.

These findings suggest that TonEBP is required for the clearance of unfolded protein aggregates and thereby increases cell survival in response to ER stress.

### 3.2. TonEBP Is Required for ER Stress-Induced Autophagosome Formation

**Figure 2:**
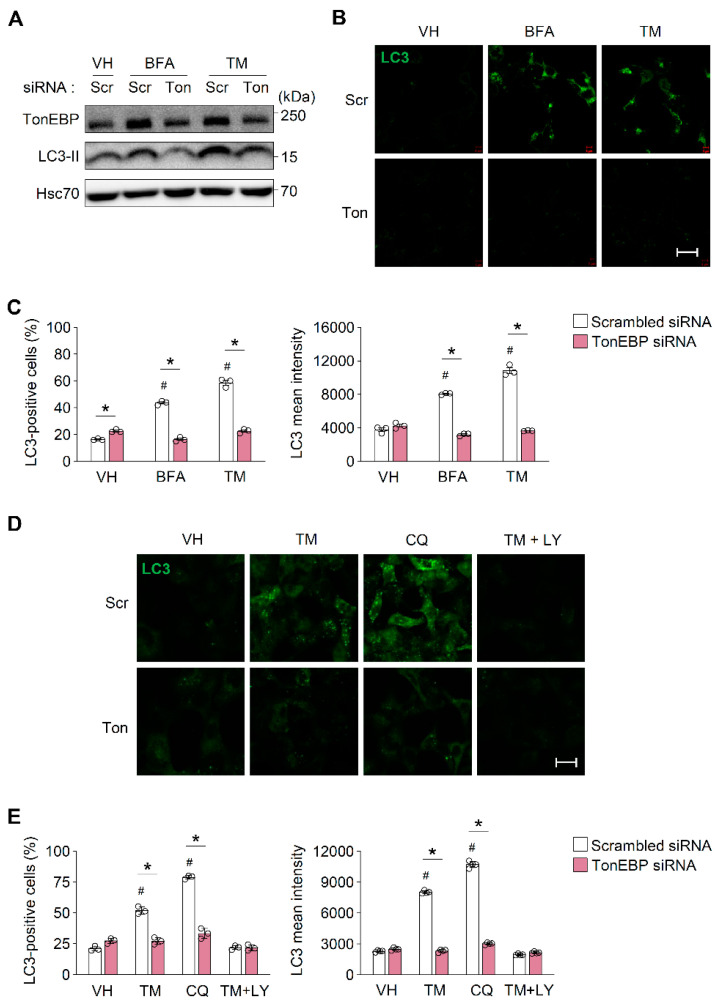
TonEBP promotes autophagy in pancreatic β cells. (**A**) MIN6-M9 cells transfected with scrambled siRNA (scr) or TonEBP-targeted siRNA (Ton) were treated for 6 h with vehicle (VH), brefeldin A (BFA; 20 μM), or tunicamycin (TM; 1 μg/mL). TonEBP, LC3-II, and Hsc70 were immunoblotted. (**B**) Cells transfected and treated as above were immunostained for LC3. (**C**) Percent of LC3 positive cells and LC3 signal intensity was measured in 150 cells from each group from (**B**). (**D**,**E**) Cells transfected with siRNA as above were pre-treated for 1 h with chloroquine (CQ; 10 μM) or LY294002 (LY; 10 μM) followed by a 4 h treatment with TM (1 μg/mL). (**D**) Cells were immunostained for LC3. (**E**) Percent of LC3 positive cells and LC3 signal intensity was measured in 50 cells from each group. Mean + SD. # *p* < 0.05 vs. scrambled siRNA-VH. * *p* < 0.05. Scale bars, 50 μm (**B**,**D**).

The induction of autophagy increases β-cell survival under ER stress by mediating the clearance of protein aggregates [37]. To elucidate the mechanism by which TonEBP increases β-cell survival under ER stress, we examined whether it stimulates autophagy in response to ER stress. During autophagosome formation, microtubule-associated protein 1 light chain 3 (LC3)-I is converted to LC3-II, which is then incorporated into the autophagosomal membrane [38]. Thus, the levels of LC3-II and LC3 correlate with the number of autophagosomes and are reliable markers of autophagosome formation [39]. A six hour treatment with ER stress inducers (20 μM BFA, and 1 μg/mL TM) markedly increased the level of LC3-II proteins in β-cells; however, this increase was markedly smaller in TonEBP-depleted cells than in control cells (Figure 2A). Furthermore, the number and intensity of LC3 puncta were higher in cells treated with ER stress inducers than in control cells, and TonEBP depletion markedly suppressed the accumulation of LC3 in response to ER stress inducers (Figure 2B,C). To further clarify the role of TonEBP during the autophagy process, we examined the effect of TonEBP depletion at the early stage (autophagosome formation) and the late stage (autophagosome-lysosome fusion) of autophagy using pharmaceutical inhibitors [40]. LY294002 (LY; 10 μM), an inhibitor of autophagosome formation, markedly suppressed TM-induced LC3 puncta (Figure 2D). On the other hand, chloroquine (CQ; 10 μM), an inhibitor of autolysosome formation, increased the accumulation of LC3, as expected from the blockade of autolysosome formation (Figure 2D). Notably, TonEBP depletion showed a similar inhibition on the accumulation of LC3 under both CQ-treated and untreated conditions (Figure 2D) indicating that TonEBP is involved in the early stage of autophagy formation. TonEBP depletion did not obviously affect the mRNA expression of the autophagy-related genes *Atg7*, *Atg14*, *p62*, and *Ulk1* (Figure S2A–D). Collectively, these data suggest that TonEBP is necessary for the induction of autophagy in β-cells.

We asked whether TonEBP mediated other forms of stress for autophagy induction. To answer this question, we examined autophagy induction by rapamycin which is a potent inducer of autophagy via the suppression of mTOR [1]. As expected, rapamycin increased the level of LC3 protein in β-cells. TonEBP depletion markedly suppressed the accumulation of LC3 in response to rapamycin (Figure S2E) indicating that TonEBP contributes to autophagy induced by multiple forms of cellular stress including, but not limited to, ER stress.

### 3.3. TonEBP Interacts with FIP200 through Its Rel-Homology Domain (RHD)

**Figure 3:**
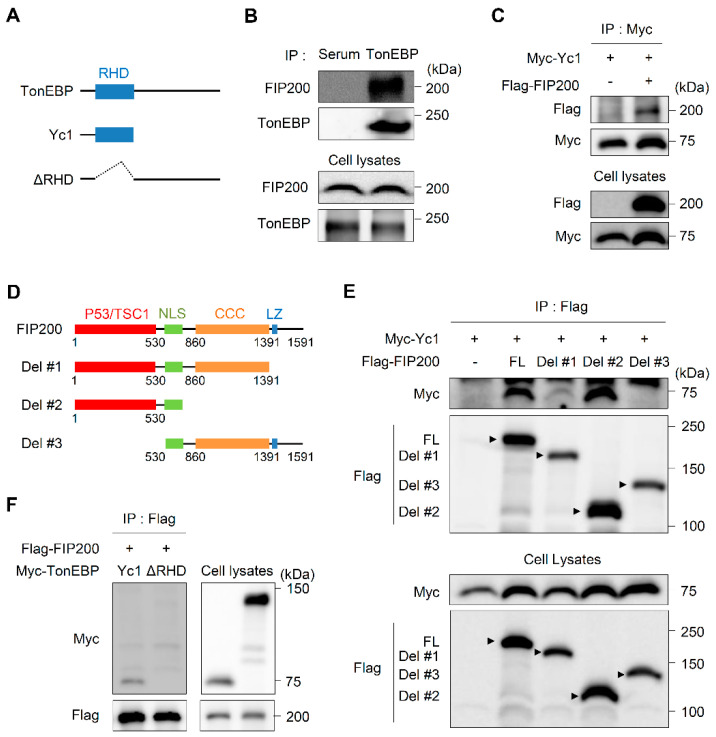
TonEBP interacts with FIP200 through the Rel-homology domain. (**A**) Domain structures of human TonEBP and deletion constructs Yc1 and ΔRel-homology domain (RHD). (**B**) MIN6-M9 cell lysates were immunoprecipitated (IP) with TonEBP antibody or normal serum as indicated. Cell lysates and precipitated proteins were immunoblotted for FIP200 and TonEBP. (**C**) Cells were transfected with plasmids expressing Myc–Yc1 without or with Flag–FIP200 as indicated. Cell lysates were immunoprecipitated with an anti-Myc antibody. Cell lysates and precipitated proteins were immunoblotted for Myc and Flag. (**D**) Domain structures of human FIP200 and deletion constructs Del #1, #2 and #3. (**E**) HEK293 cells were transfected with plasmids expressing Myc-Yc1 together with Flag-tagged FIP200 (FL), Del #1, Del #2 or Del #3 as indicated. Cell lysates were immunoprecipitated with an anti-Flag antibody. Cell lysates and precipitated proteins were immunoblotted for Myc and Flag. (**F**) HEK293 cells were transfected with a plasmid expressing Flag–FIP200 together with a plasmid expressing Myc–Yc1 or Myc–ΔRHD as indicated.

Next, we investigated the mechanism by which TonEBP functions in ER stress-induced autophagy. To this end, we analyzed proteins that interacted with an N-terminal truncated form of TonEBP containing the intact RHD (Yc1) (Figure 3A) by performing a tandem affinity purification [31]. FIP200, a ULK-interacting protein that is required for autophagosome formation in mammalian cells [41], was a top hit (Figure S3). We performed reciprocal co-immunoprecipitation experiments to confirm the interaction between TonEBP and FIP200. Experiments using both endogenous (Figure 3B) and overexpressed (Figure 3C) proteins revealed that TonEBP pulled down FIP200 and vice versa.

To define which sites mediate the TonEBP–FIP200 interaction, we generated constructs that expressed several TonEBP (Figure 3A) and FIP200 (Figure 3D) mutant proteins. To identify which structural elements of FIP200 are important for its interaction with TonEBP, cells were transfected with constructs that expressed Yc1 and full-length FIP200 or a deletion mutant. Yc1 was co-immunoprecipitated by full-length FIP200, Del #1 (ΔLz), and Del #2 (ΔCCC and ΔLz). However, the deletion of the p53/TSC1 domain (Del #3) abolished the interaction with Yc1, demonstrating that this domain of FIP200 is required for its interaction with TonEBP (Figure 3E). In addition, a TonEBP mutant lacking the RHD (ΔRHD) did not interact with FIP200 (Figure 3F), indicating that this domain of TonEBP is essential for its interaction with FIP200. Collectively, these data suggest that the RHD of TonEBP and the p53/TSC1 domain of FIP200 mediate the interaction of these two proteins.

**Figure 4:**
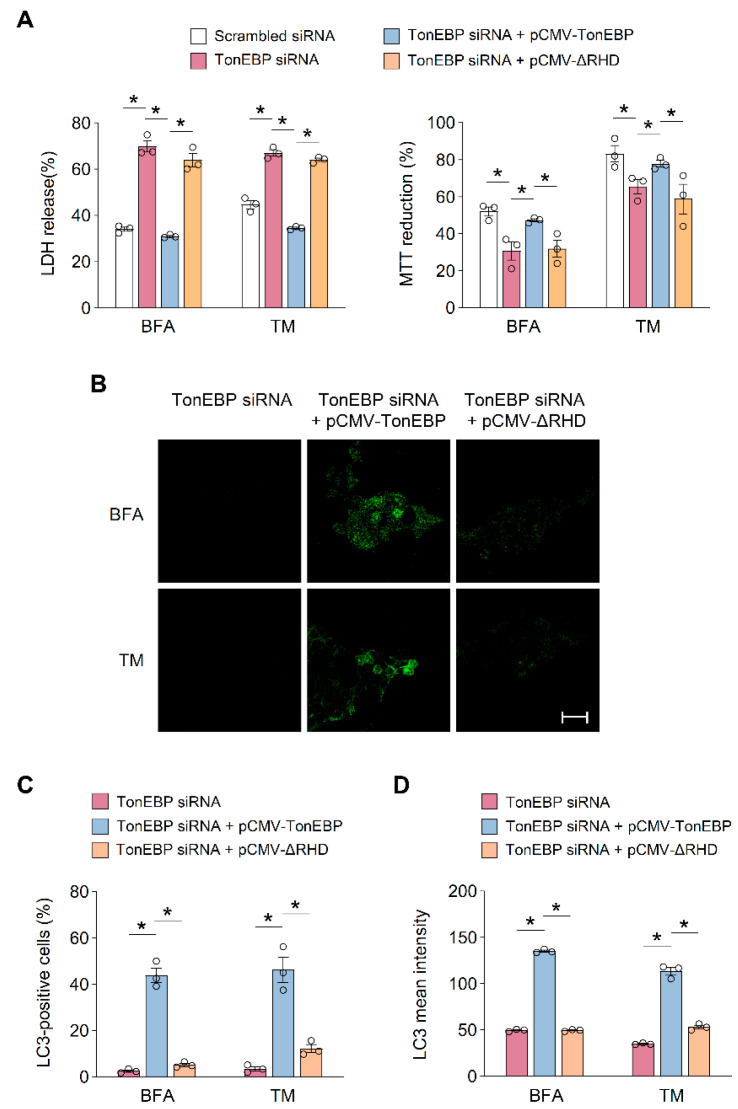
RHD of TonEBP is required for endoplasmic reticulum (ER) stress-induced autophagy. (**A**–**D**) MIN6-M9 cells were transfected with scrambled or TonEBP-targeted siRNA followed by a second transfection with a plasmid expressing TonEBP or ΔRHD as indicated. (**A**) Cell viability was assessed by the LDH release (**A**) and MTT reduction (**B**) after a 24 h treatment of brefeldin A (BFA; 20 μM) or tunicamycin (TM; 1 μg/mL). (**B**) LC3 was detected with immunostaining. (**C**) Percent of LC3 positive cells was measured in 150 cells from each group. (**D**) LC3 signal intensity was measured in 150 cells from each group. Data (mean + SD) were from three independent experiments (*n* = 3) each with more than three replicates. * *p* < 0.05 ((**A**,**C**,**D**); Two-way ANOVA with Tukey’s post-hoc test). Scale bars, 50 μm (**B**).

Based on these results, we hypothesized that the RHD of TonEBP plays an important role in cell viability and the activation of autophagy under ER stress. To investigate this, we performed rescue experiments in which wild-type TonEBP or a mutant lacking the RHD was expressed. The siRNA-mediated knockdown of TonEBP decreased cell viability over a 24 h treatment with the ER stress inducers BFA (20 μM) and TM (1 μg/mL). The reduction in cell viability by TonEBP depletion upon treatment with each of the two ER stress inducers was rescued by the expression of wild-type TonEBP, but not by the expression of the TonEBP mutant lacking the RHD (Figure 4A). Furthermore, the accumulation of LC3 in TonEBP-depleted cells under ER stress was enhanced by the expression of wild-type TonEBP but was unaffected by the expression of the TonEBP mutant lacking the RHD (Figure 4B–D).

Taken together, these data suggest that the RHD of TonEBP is required for its interaction with FIP200, activation of autophagy, and cell survival under ER stress.

### 3.4. ER Stress Enhances the Stability of TonEBP Proteins

**Figure 5:**
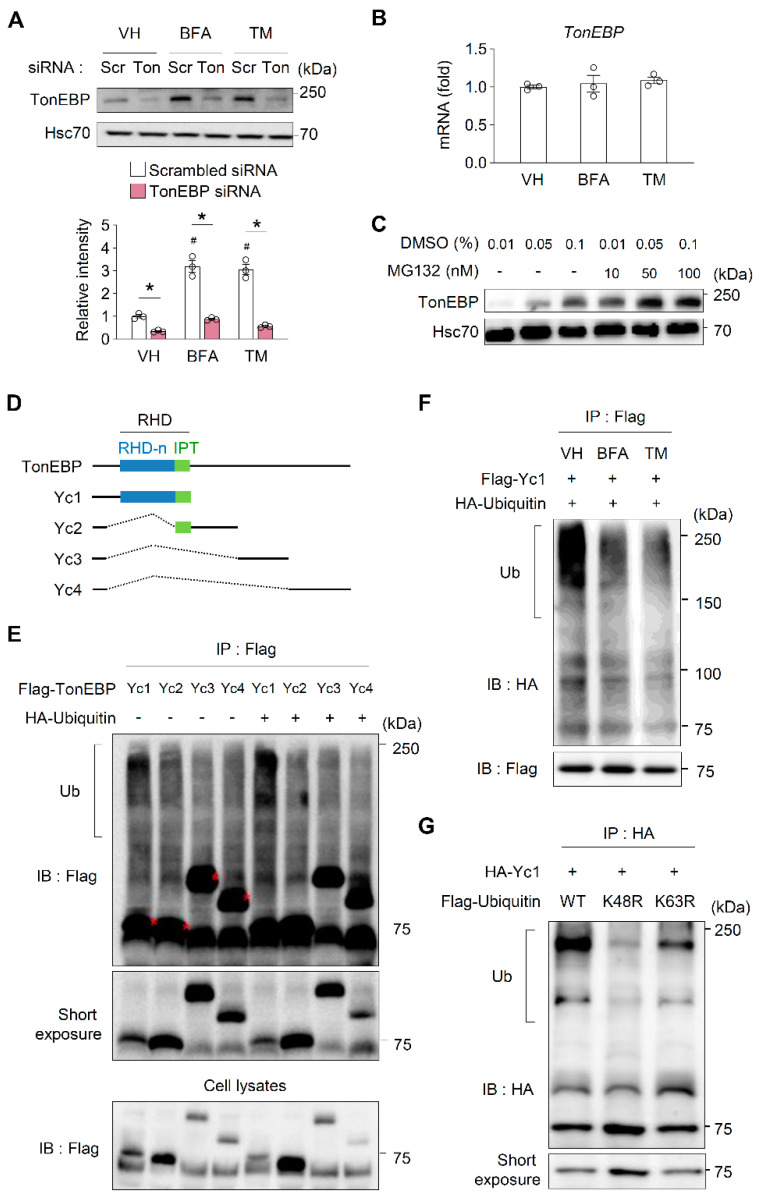
ER stress dramatically increases TonEBP protein stability. (**A**) MIN6-M9 cells transfected with scrambled siRNA (scr) or TonEBP-targeted siRNA (Ton) were treated for 4 h with vehicle (VH), brefeldin A (BFA; 20 μM), or tunicamycin (TM; 1 μg/mL) as indicated. TonEBP and Hsc70 were immunoblotted. Data (mean + SD) were from three independent experiments (*n* = 3) each with more than three replicates. # *p* < 0.05 vs. scrambled siRNA-VH. * *p* < 0.05 (one-way ANOVA). (**B**) Cells were treated for 6 h with the same agents as in (**A**). TonEBP mRNA was measured by RT Q-PCR. Mean + SD, *n* = 4. (**C**) Cells were treated for 4 h with 10–100 nM MG132 or 0.01–0.1% of DMSO (vehicle) as indicated. (**D**) Structures of human TonEBP and their serial deletion constructs Yc1, Yc2, Yc3 and Yc4. (**E**) HEK293 cells were transfected with plasmid expressing Flag-tagged Yc1, Yc2, Yc3 or Yc4 alone or in combination with another plasmid expressing HA-ubiquitin as indicated. Proteins immunoprecipitated with an anti-Flag antibody were immunoblotted with the same antibody. (**F**) Cells were transfected with two plasmids expressing Flag–Yc1 and HA–ubiquitin. Cells were the treated for 1 h with agents shown in (**A**). Proteins immunoprecipitated with an anti-Flag antibody were immunoblotted with an anti-HA antibody. (**G**) HEK293 cells were transfected with HA–Yc1 together with Flag–ubiquitin (WT), Flag–ubiquitin K48R mutant (K48R) or Flag–ubiquitin K48R mutant (K63R). Proteins immunoprecipitated with an anti-HA antibody were immunoblotted with the same antibody. (**E**–**G**) Ub, ubiquitinated proteins.

The contribution of TonEBP to cell survival under ER stress (Figure 1) led us to examine whether ER stress influences its expression. A treatment of of four hours with the ER stress inducers (20 μM of BFA and 1 μg/mL of TM) increased the protein expression of TonEBP in β-cells (Figure 5A). However, the mRNA expression of TonEBP was unaffected (Figure 5B), demonstrating that TonEBP is regulated post-translationally in response to ER stress. Consistently, the treatment with MG132, a potent proteasome inhibitor, dose-dependently (10–100 nM) increased the level of TonEBP proteins in β-cells (Figure 5C), suggesting that the ubiquitin–proteasome pathway contributes to the stability of TonEBP. To investigate the ubiquitination of TonEBP, we transfected HEK293 cells with constructs expressing various deletion mutants of TonEBP (Figure 5D). Only cells expressing Yc1 displayed ubiquitination and this was enhanced by the ectopic expression of ubiquitin (Figure 5E), suggesting that the RHD of TonEBP is the main ubiquitination target. We next examined the ubiquitination of TonEBP under ER stress. A one-hour treatment with the ER stress inducers (20 μM of BFA and 1 μg/mL of TM) reduced the ubiquitination of TonEBP, indicating that the stability of TonEBP proteins is enhanced under ER stress (Figure 5F). Recent studies have shown that different linkage types of the ubiquitin chain elicit different effects on substrates [42,43]. The K48-linked ubiquitin chain mediates the proteasomal degradation of substrates, whereas the K63-linked polyubiquitin chain is involved in the regulation of the activities, localizations, and binding partners of substrates. Yc1 was ubiquitinated when wild-type ubiquitin was expressed; however, its ubiquitination was markedly decreased when the K48R or K63R ubiquitin mutant was expressed (Figure 4G). Collectively, these data suggest that the protein stability of TonEBP is enhanced under ER stress due to a reduction in its degradation via the ubiquitin–proteasome pathway and that this increases cell survival.

### 3.5. Deletion of TonEBP in Pancreatic Endocrine Progenitor Cells Perturbs Glucose Homeostasis

**Figure 6:**
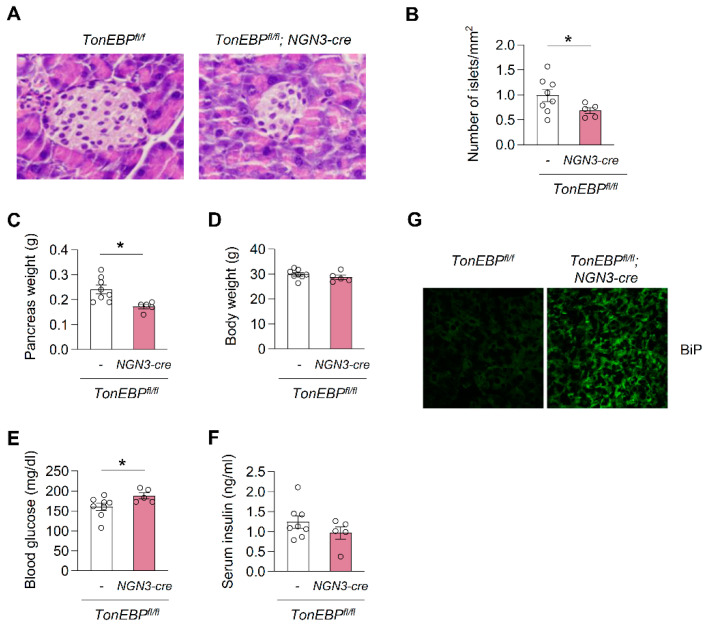
Smaller and fewer pancreatic islets in association with hyperglycemia in animals with islet-specific TonEBP deficiency. (**A**) Representative hematoxylin and eosin (H&E) stained images of pancreatic islets in *TonEBP^fl/fl^; NGN3 cre^+/−^* mice and their *TonEBP^fl/fl^* littermates. (**B**) Number of pancreatic islets per mm^2^ of tissues sections are shown in (**A**). Pancreas weight (**C**), body weight (**D**), plasma glucose (**E**) and plasma insulin (**F**) were analyzed from 14 week old animals: *TonEBP^fl/fl^; NGN3 cre^+/−^* mice (blue, *n* = 5) and their *TonEBP^fl/f^* littermates (red, *n* = 8). (**G**) Representative fluorescence microscopic images showing BiP from *TonEBP^fl/fl^; NGN3 cre^+/−^* mice and their *TonEBP^fl/f^* littermates. *n* represents the number of biologically independent animals. Mean + SE, * *p* < 0.05 ((**B**–**F**); unpaired *t*-test).

We next sought to determine the impact of TonEBP deficiency on pancreatic homeostasis in vivo. To this end, we generated *TonEBP^fl/fl^ NGN3-cre* mice in which TonEBP was deleted in pancreatic endocrine progenitor cells using the Cre-lox system (*TonEBP^fl/fl^*; *neurogenin 3 promoter driven-Cre*). Floxed TonEBP mice that did not express Cre recombinase (*TonEBP^fl/f^* alone) were used as a control. The deletion of TonEBP in pancreatic endocrine progenitor cells significantly decreased the size of islets (Figure 6A), number of islets (Figure 6B), and pancreas weight (Figure 6C), but did not affect body weight (Figure 6D). Consistently, the serum glucose level was higher in *TonEBP^fl/fl^ NGN3-cre* mice than in control mice (Figure 6E), but the serum insulin level was unchanged (Figure 6F), suggesting that TonEBP deletion in pancreatic endocrine progenitor cells perturbs glucose homeostasis. We also examined ER stress and autophagy in the pancreas by performing immunofluorescence staining for BiP, respectively. The accumulation of BiP was greater in *TonEBP^fl/fl^ NGN3-cre* mice than in control mice (Figure 6G). Taken together, these data suggest that TonEBP is required for homeostasis in the pancreas via the modulation of ER stress and autophagy. However, these findings do not exclude the possibility that TonEBP is involved in pancreas development and this requires further investigation.

## 4. Discussion

**Figure 7:**
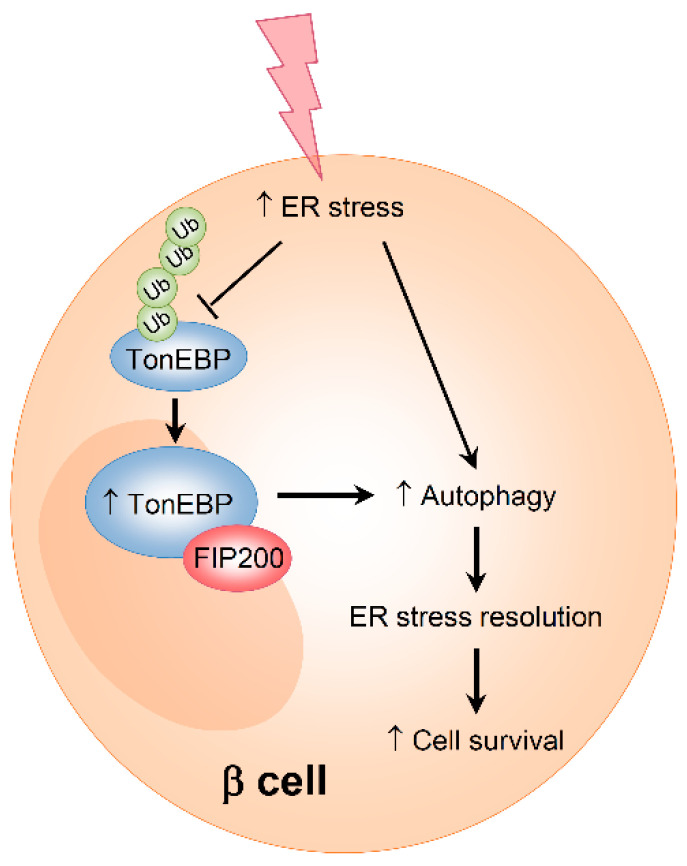
Model for ER stress-induced stabilization of TonEBP and autophagy formation in pancreatic β cells. TonEBP is deubiquitinated in response to ER stress. As a result, TonEBP is stabilized and interacts with FIP200 leading to the initiation of autophagy formation which prevents cell death.

TonEBP is a pleiotropic stress protein that mediates both protective and pathological cellular responses in a stress- and cell type-dependent manner [17]. The primary finding of this study is that TonEBP protects pancreatic β-cells against ER stress. We showed that (a) the protein expression of TonEBP in β-cells is elevated in response to ER stress due to its increased protein stability, (b) TonEBP increases β-cell survival under ER stress, (c) TonEBP inhibits the accumulation of ER stress-related proteins, and (d) TonEBP attenuates ER stress-associated cell death via the regulation of autophagy (Figure 7). These findings provide new insights into the role of TonEBP in the context of ER stress and autophagy.

The ER stress response and autophagy are important cellular responses that determine cell fate (survival and death) [1,2,3,8,9] and whose dysregulation is implicated in various human diseases [4,44,45,46]. Persistent ER stress results in the accumulation of misfolded and/or aggregated proteins in the ER that are a hallmark of protein conformational disorders [1,2]. Thus, the cellular protein quality control machinery is critical for the cellular and organismal physiology. The aggregated proteins and dysfunctional organelles are removed by autophagy-mediated lysosomal degradation. Misfolded proteins without aggregation can be restored by molecular chaperones [47] or removed by proteasomal degradation [10]. Emerging evidence indicates that there is crosstalk and coordination between the three protein degradation systems [47,48]. Autophagy can be stimulated as a secondary response to multiple types of cellular stress, including ER stress, in order to alleviate stress [11,12,49]. Autophagy functions in physiology and pathophysiology, and thus the finding that it is regulated by TonEBP, are particularly important. Autophagy occurs at a basal rate in a range of normal human physiological processes to maintain cellular homeostasis [50], and is essential for development and differentiation [50,51]. More importantly, autophagy is involved in various human disorders. The induction of autophagy protects against aging [52], metabolic syndrome [53,54,55], neurodegenerative diseases [56,57,58], infectious diseases [59,60,61], and some cancers [62,63]. Conversely, autophagy acts as a pro-survival pathway in certain cancers [64,65,66]. Given these observations, approaches to activate or inhibit autophagy are currently receiving considerable attention as potential therapeutic strategies for diverse diseases. The identification of TonEBP as a novel regulator of autophagy provides a significant insight into the mechanisms underlying the regulation of autophagy and autophagy-modulating strategies.

Although the molecular mechanism linking ER stress and autophagy remains to be fully elucidated, multiple signaling pathways are reportedly involved in the crosstalk between autophagy and ER stress [3,67]. Autophagy is tightly regulated by mTOR, the ULK1–ATG13–FIP200 complex, and ATG, indicating that significant crosstalk occurs between signaling pathways [44,45]. The ULK1–ATG13–FIP200 complex is essential for the initiation of autophagy [68]. Here, we showed that TonEBP interacts with FIP200, which is essential for autophagosome formation [69], via its RHD; this domain of TonEBP is necessary for its function in autophagy and β-cell survival under ER stress (Figure 4A–D). We hypothesize that TonEBP functions in ER stress-induced autophagy via its interaction with FIP200; however, further studies are required to verify this. The finding that TonEBP interacts with FIP200 is of great interest because FIP200 has distinct roles in cellular homeostasis and disease pathogenesis in different cell types. The depletion of FIP200 in neurons [70] and hematopoietic stem cells [71] leads to phenotypic defects associated with the suppression of autophagy. The non-autophagic functions of FIP200 are crucial during embryogenesis [72]. FIP200 also inhibits the progression of several types of cancer [73]. By contrast, another study showed that the function of FIP200 in autophagy supports tumor cell growth [74], suggesting that FIP200 is a potential target for cancer therapy. FIP200 regulates several intracellular signaling pathways via interactions with other proteins [75,76,77,78], and we identified TonEBP as an intracellular binding partner of FIP200. It will be interesting to investigate the impact of the TonEBP–FIP200 interaction on autophagy and other cellular responses in future studies.

The primary finding of this study is that TonEBP protects β-cells against ER stress. Although TonEBP has both protective and pathological effects in a stress- and cell type-dependent manner, the interpretation of this result is complicated by the previous findings that islet autoimmunity in humans is associated with an increased expression of TonEBP [23] and that the blood glucose level in mice with global TonEBP haplo-deficiencies is comparable with that in their wild-type littermates [79]. However, there is a possible explanation for these discrepant findings. Pancreatic islets contain numerous other cell types (e.g., immune cells, vascular cells, and stromal cells) in addition to endocrine cells [80]. The survival and death of β-cells might involve cell–cell crosstalk through the coordination of multiple processes as well as intrinsic pathways. Pro-inflammatory M1 macrophages, CD4^+^ T lymphocytes, and CD8^+^ cytotoxic T-cells are considered to be the major cell types that promote the development of type 1 DM [81,82], while M2 macrophages, which function in wound healing and tissue remodeling, promote β-cell proliferation by inducing crosstalk among different cell types [83]. Notably, a depletion of TonEBP in macrophages suppresses the polarization of M1 macrophages [30,84] and activates the polarization of M2 macrophages [29,85]. More importantly, the downregulation of TonEBP attenuates pathological CD4^+^ T cell differentiation and autoimmunity [19,86]. Based on these findings, we believe that the loss of β-cells induced by a TonEBP deficiency can be rescued by enhanced protective or homeostatic functions in immune cells lacking TonEBP.

The level of TonEBP is elevated in response to various stresses [17]. The upregulation of TonEBP protein under stress is paralleled by an increase in *TonEBP* mRNA [17]. The *TonEBP* gene promoter has not been defined and thus it remains unknown whether it is regulated by stress. In many cases, the downregulation of microRNAs targeting *TonEBP* mRNA leads to the upregulation of this mRNA [17]. Here, we demonstrated that the level of TonEBP is regulated by its protein stability. We showed that ER stress increases the level of TonEBP in β-cells (Figure 5A) and that this is due to an increase in the protein stability of TonEBP owing to a reduction in its ubiquitin-mediated proteasomal degradation. Additionally, we showed that both K48- and K63-linked ubiquitin chains, which are the two most abundant chain types, are involved in the ubiquitination of TonEBP. To the best of our knowledge, this is the first study to show that the level of TonEBP protein is influenced by its stability. Our previous study demonstrated that TonEBP interacts with the E3 ubiquitin ligase SHPRH and the deubiquitinase USP1, and that this correlates with PCNA polyubiquitination in response to DNA damage [31]. Based on these findings, we speculate that SHPRH and USP1 mediate the ubiquitination and deubiquitination of TonEBP. However, further studies focusing on the mechanism that regulates TonEBP ubiquitination are needed to verify this hypothesis.

In summary, our results reveal that TonEBP is upregulated and increases β-cell survival by enhancing autophagy under ER stress. These findings demonstrate the previously unknown role of TonEBP in the ER stress response and autophagy.

## Supplementary Materials

The following are available online at https://www.mdpi.com/2073-4409/9/9/1928/s1, Figure S1: TonEBP promotes autophagy without changes in ER stress-related protein and mRNA expression, Figure S2: TonEBP promotes autophagy without changes in ATG7, ATG14, p62 and ULK1 mRNA, Figure S3: TonEBP-interacting proteins related to autophagy initiation, Table S1: Primers used for real time PCR.
