# Supplementary material for: TonEBP Promotes β-Cell Survival under ER Stress by Enhancing Autophagy
Source: Cells. 2020 Aug 20;9(9):1928. doi: 10.3390/cells9091928 (PMC7563687; doi:10.3390/cells9091928)
Supplement: Supplementary file 1 [file cells-09-01928-s001.pdf]

Supplementary Information

TonEBP increases β-cell survival under ER stress by enhancing autophagy

Kang et al.

Supplementary Methods

RNA isolation and real-time PCR

Total RNA from MIN6-M9 cells was isolated using the TRIzol reagent (Invitrogen) according to the manufacturer's instructions. cDNA was synthesized by M-MLV reverse transcriptase (Promega, Madison, WI, USA). After reverse transcription, real-time PCR was performed using SYBR Green I Master and LightCycler 480 II (Roche). Measured cycle threshold values were normalized for the cyclophilin A reference gene, and they were expressed as fold change over control samples. The primers are described in Supplementary Table 1.

Supplementary figure legends

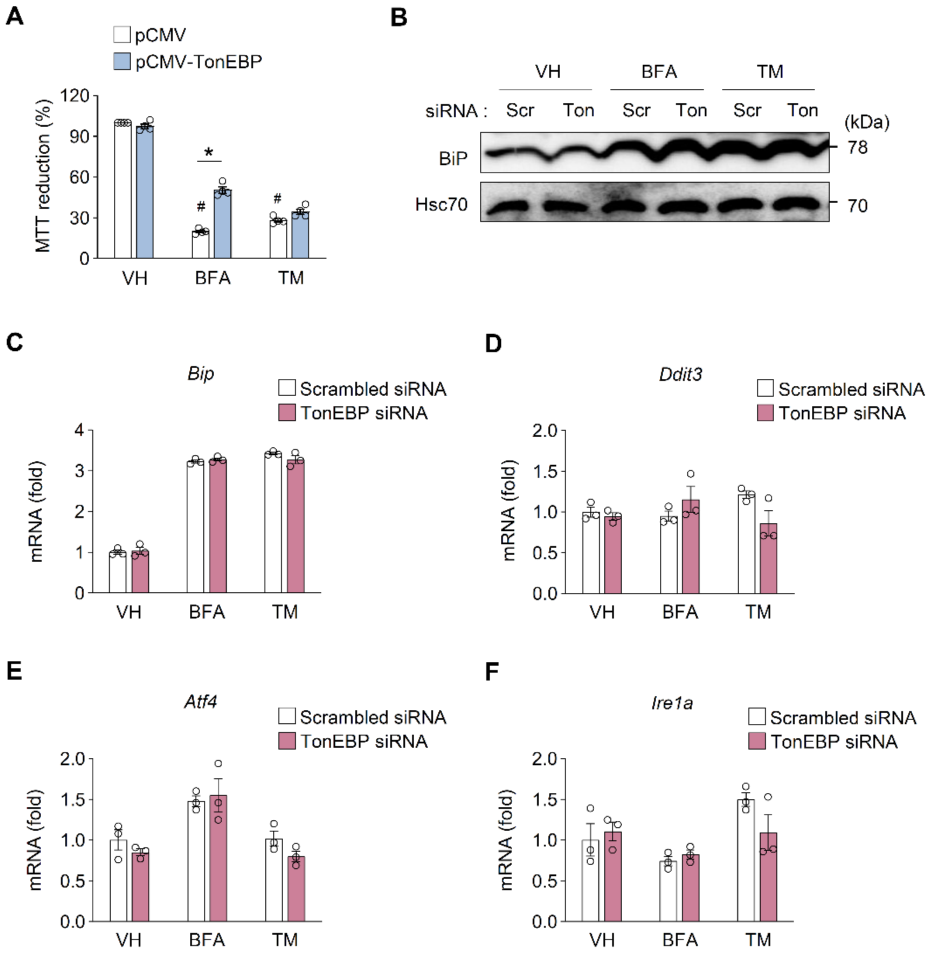

Figure S1. TonEBP promotes autophagy without changes in ER stress-related protein and mRNA expression. (**A**) MIN6-M9 cells were transfected with plasmids expressing TonEBP or vector alone (pCMV) and then treated with brefeldin A (BFA; 20 μM) or tunicamycin (TM; 1 μg/ml). Cell viability calculated in %. Mean + SD. # $p < 0.05$ vs pCMV-VH. *$p < 0.05$. (**B-F**) MIN6-M9 cells were transfected with scrambled siRNA (scr) or TonEBP-targeted siRNA (Ton) and then treated with brefeldin A (BFA; 10μM) or tunicamycin (TM; 1μg/ml) for 4 h. (**B**) The cells were immunoblotted for BiP and Hsc70. (**C**

- F) mRNA expression for *Bip* (**C**), *Ddit3* (**D**), *Atf4* (**E**) and *Ire1a* (**F**) was examined by RT Q-PCR. Mean + SD, *n* = 3. *p < 0.05 (unpaired t-test).

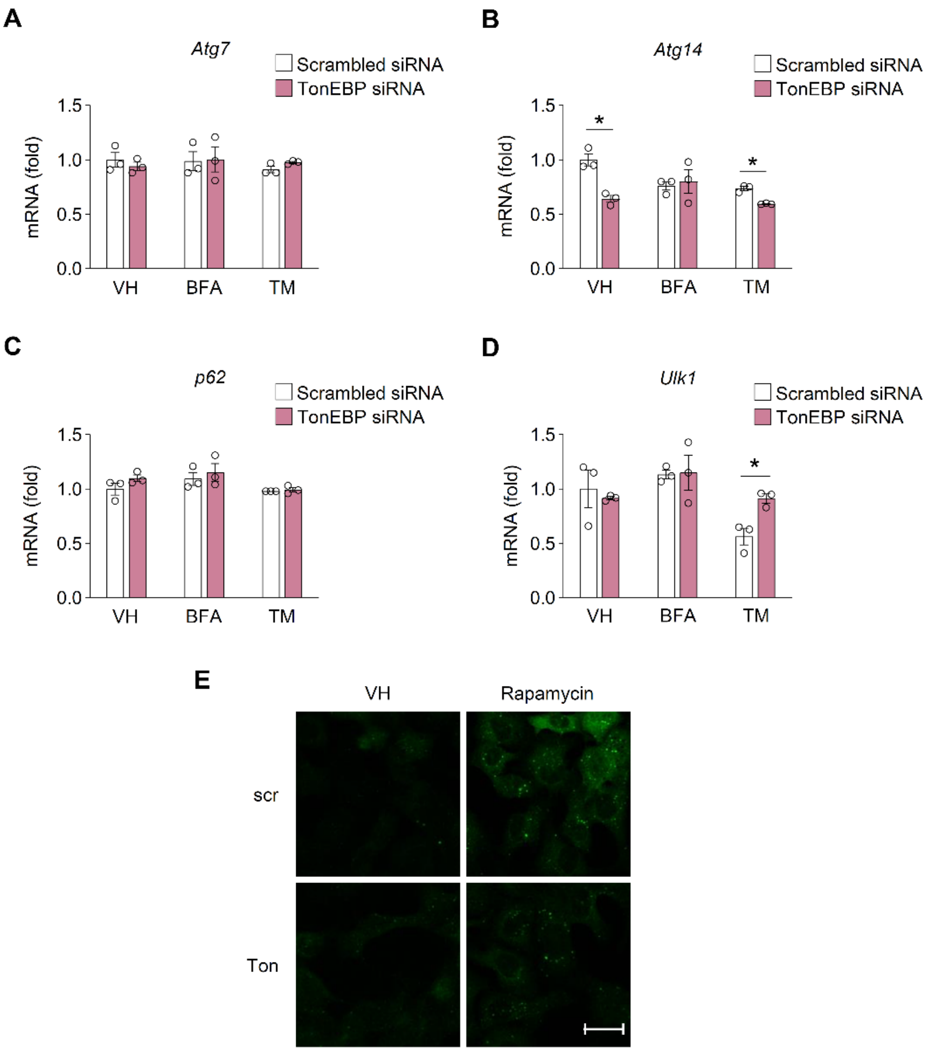

Figure S2. TonEBP promotes autophagy without changes in ATG7, ATG14, p62 and ULK1 mRNA. (**A–D**) MIN6-M9 cells were transfected with scrambled-siRNA or TonEBP-targeted siRNA. Cells were treated with brefeldin A (BFA; 20 μM) or tunicamycin (TM; 1 μg/ml) for 4 h and mRNA expression for *Atg7* (**A**), *Atg14* (**B**), *p62* (**C**) and *Ulk1* (**D**) was examined by RT Q-PCR. Mean + SD, *n* = 4. *p < 0.05 (unpaired t-test). (**E**) Cells transfected with scrambled siRNA (scr) or TonEBP-targeted siRNA (Ton) were treated for 6 h with vehicle (VH) or rapamycin (1 μM). Cells were then immunostained for LC3.

Number	Gene (Fold change > 2)
1	ATG4B
2	ATG7
3	CTSD
4	MTOR
5	PAFAH1B2
6	RB1CC1 (FIP200)
7	S100A8
8	S100A9
9	SNAP29
10	USP13

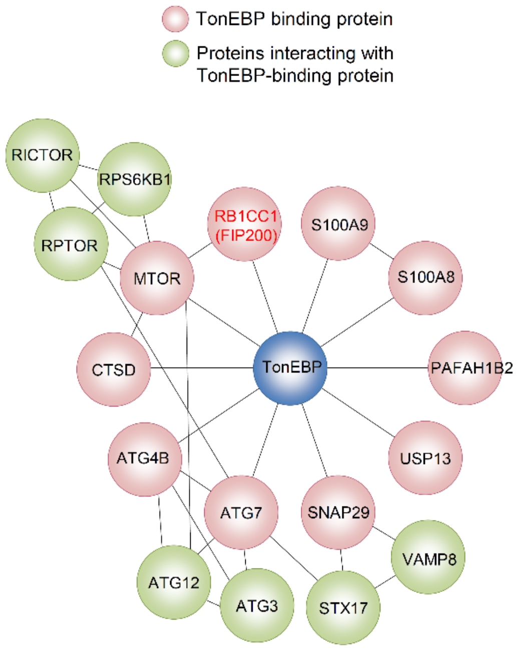

Figure S3. TonEBP-interacting proteins related to autophagy initiation.Yc1 of TonEBP was used to detect TonEBP-binding proteins (1). Among >460 interacting proteins, those proteins known to be involved in autophagy initiation are shown in red. Proteins predicted to interact with TonEBP-binding proteins (green) were identified using STRING (https://string-db.org/).

Table S1. Primers used for real time PCR.

Species	Gene	Forward primer (5'-3')	Reverse primer (5'-3')
Mouse	*TonEBP*	AAGCAGCCACCACCAAACATGA	AAATTGCATGGGCTGCTGCT
	BiP	TGCAGCAGGACATCAAGTTC	TACGCCTCAGCAGTCTCCTT
	Ddit3	AAGATGAGCGGGTGGCAGCG	GCACGTGGACCAGGTTCTGCT
	Atf4	GAGCTTCCTGAACAGCGAAGTG	TGGCCACCTCCAGATAGTCATC
	Ire1a	TGTGGTCAAGATGGACTGG	GAAGCGGGAAGTGAAGTAGC
	Atg7	ATGCCAGGACACCCTGTGAACTTC	ACATCATTGCAGAAGTAGCAGCCA
	Atg14	TGTACCTGGTCAGTCCAAGCTC	CAGGTCGGTTTCTTCATCGCTG
	p62	TGTGGAACATGGAGGGAAGAG	TGTGCCTGTGCTGGAACTTTC
	Ulk1	GCAGCAAAGACTCCTGTGACAC	CCACTACACAGCAGGCTATCAG

References

1. Kang, H.J.; Park, H.; Yoo, E.J.; Lee, J.H.; Choi, S.Y.; Lee-Kwon, W.; Lee, K.Y.; Hur, J.H.; Seo, J.K.; Ra, J.S., et al. TonEBP Regulates PCNA Polyubiquitination in Response to DNA Damage through Interaction with SHPRH and USP1. *iScience* **2019**, *19*, 177-190, doi:10.1016/j.isci.2019.07.021.
